# Cheap Dentistry and Its Tendencies

**Published:** 1859-08

**Authors:** John R. Allen

**Affiliations:** N. Y.


### Selections.

### CHEAP DENTISTRY AND ITS TENDENCIES.

BY PROF. JOHN R. ALLEN, N. Y.

There is a great tendency at the present age to cheapen every manufactured article. The manufacturer, in order to promote his own pecuniary interests, must sacrifice, in a greater or a less degree, the interests of his patrons, for he employs inferior materials with which he constructs his fabrics. He also bestows less labor upon them to diminish the expense, and forms them more rudely, which requires a less degree of talent and skill. Hence we have many products like the Frenchman's, whose razors were not made to shave with, but to sell, and the buyer as well as the article purchased is generally sold. The dental profession is also tinctured with the same mania, and there are those who study more how to cheapen and lessen the value of their operations, than to increase their utility by superior excellence.

This distructive policy can be tolerated better in almost any other sphere than in dental practice; for if cheap dentistry were to prevail, the tendency with the profession would be retrogressive. Science and art would be diverted from dentistry into other channels, for those who possess them would naturally seek more lucrative vocations.

The thorough education, the requisite amount of taste and talent, of art and science, which are necessary for a good practical dentist to possess, would yield him a better remuneration if employed in other branches of business; for it should be remembered that dental practice cannot be enlarged like that of a mechanic, a merchant, or a manufacturer, who can employ fifty, a hundred, or five hundred hands, while the operations of a dentist are limited to a few fingers; and yet those fingers, if skilled in manipulation, and directed by a competent mind, may do as much to ameliorate the condition of man as the mechanic or manufacturer, and justice would claim for him at least an equal reward.

Aside from charitable considerations, when dental services are rendered for a less sum than a fair compensation, they come under the head of cheap dentistry, which is considered synonymous with *poor* dentistry, and poor dentistry tends to create distrust and destroy public confidence, which would place dental surgery in comparative obscurity should it become universal. The cheap dentist is ever regarded as being below par in his profession by the community where he lives, for let him place ever so low an estimate upon his services, the public will *mark* him still lower, and he is looked upon according to the level which he seeks; and after he has once taken his position and announced his rank through the medium of the public press, he will find it extremely difficult to change his grade should he subsequently wish to do so, for his *cast* is *fixed* and stamped with his own seal.

It is not the *cheapest* but the *best* dentists who are sought for by the major part of those who require dental aid; and the price is a secondary consideration. They require our best services, for which they are willing to pay a fair compensation, unless they have been misled by those who underrate the value and importance of dental operations when skilfully performed.

No, rather than pull down dental practice by cheapening and rendering it less valuable, let us strive to enhance its value, by aiming at a still higher degree of perfection; for it is more honerable and more lucrative to occupy an elevated position in the profession, than a low, grovelling one, that will ever be looked down upon with distrust.

